# [Expression of Concern] Ultraviolet exposure of melanoma cells induces fibroblast activation protein-α in fibroblasts: Implications for melanoma invasion

**DOI:** 10.3892/ijo.2026.5940

**Published:** 2026-09-16

**Authors:** Petra Wäster, Inger Rosdahl, Brendan F. Gilmore, Oliver Seifert

Int J Oncol 39: 193-202, 2011; DOI: 10.3892/ijo.2011.1002

Following the publication of the above paper, it was drawn to the Editor's attention by a concerned reader that the control β-actin blots shown in Fig. 1D and G on p. 195 appeared to be very similar, albeit with rotation of one set of the blots through 180°, and with resizing of the bands. Similarly, certain of the β-actin protein bands shown in Fig. 1G were strikingly similar to the control β-actin protein bands shown in Fig. 5A on p. 200, suggesting that data in these figures may have been assembled incorrectly.

The authors were contacted by the Editorial Office to offer an explanation for the apparent anomalies in the presentation of the data in this paper, and they have subsequently replied to explain that, unfortunately, they are unable to re-analyze the original data or repeat the relevant experiments to address these concerns, as the study was conducted many years ago, the data are no longer available, and the authors are no longer active in this field of research. The authors would therefore advise readers of the above article to interpret the western blots shown in Figs. 1 and 5 with caution. Owing to the fact that the Editorial Office has been made aware of potential issues surrounding the scientific integrity of certain of the data this paper, we are issuing an Expression of Concern to notify readers of this potential problem.

